# Quantifying the biochemical state of knee cartilage in response to running using T1rho magnetic resonance imaging

**DOI:** 10.1038/s41598-020-58573-8

**Published:** 2020-02-05

**Authors:** Lauren N. Heckelman, Wyatt A. R. Smith, Alexie D. Riofrio, Emily N. Vinson, Amber T. Collins, Olivia R. Gwynn, Gangadhar M. Utturkar, Adam P. Goode, Charles E. Spritzer, Louis E. DeFrate

**Affiliations:** 1 Department of Orthopaedic Surgery, Duke University School of Medicine, Durham, USA; 2 Department of Biomedical Engineering, Pratt School of Engineering, Duke University, Durham, USA; 3 Department of Radiology, Duke University School of Medicine, Durham, USA; 4 Department of Population Health Sciences, Duke University School of Medicine, Durham, USA; 5 Duke Clinical Research Institute, Durham, USA; 6 Department of Mechanical Engineering & Materials Science, Pratt School of Engineering, Duke University, Durham, USA

**Keywords:** Cartilage, Biomedical engineering, Mechanical engineering

## Abstract

Roughly 20% of Americans run annually, yet how this exercise influences knee cartilage health is poorly understood. To address this question, quantitative magnetic resonance imaging (MRI) can be used to infer the biochemical state of cartilage. Specifically, T1rho relaxation times are inversely related to the proteoglycan concentration in cartilage. In this study, T1rho MRI was performed on the dominant knee of eight asymptomatic, male runners before, immediately after, and 24 hours after running 3 and 10 miles. Overall, (mean ± SEM) patellar, tibial, and femoral cartilage T1rho relaxation times significantly decreased immediately after running 3 (65 ± 3 ms to 62 ± 3 ms; p = 0.04) and 10 (69 ± 4 ms to 62 ± 3 ms; p < 0.001) miles. No significant differences between pre-exercise and recovery T1rho values were observed for either distance (3 mile: p = 0.8; 10 mile: p = 0.08). Percent decreases in T1rho relaxation times were significantly larger following 10 mile runs as compared to 3 mile runs (11 ± 1% vs. 4 ± 1%; p = 0.02). This data suggests that alterations to the relative proteoglycan concentration of knee cartilage due to water flow are mitigated within 24 hours of running up to 10 miles. This information may inform safe exercise and recovery protocols in asymptomatic male runners by characterizing running-induced changes in knee cartilage composition.

## Introduction

Approximately 65 million individuals in the United States report jogging or running within the last 12 months^1^. Despite the widespread popularity of this activity, there is a paucity of data regarding the impact of running on knee cartilage health, especially in the long-term^2–4^. Previous studies have investigated how running alters the biochemical state of knee cartilage using magnetic resonance imaging (MRI) techniques^3,5–8^. However, most of these investigations were designed to probe the effects of marathon running on the cartilage^3,5,7^, while less than 1% of runners successfully finished a marathon in 2016^9^. Thus, at present, little is known regarding how knee cartilage responds to loads incurred during running more moderate recreational distances.

It is well-established that some amount of mechanical loading is essential for maintaining cartilage health^10–19^. However, the optimal timing and intensity of this loading has not yet been determined. Furthermore, there is some evidence to suggest that repetitive long-distance running may lead to structural changes in the cartilage, including changes in proteoglycan concentration^3^. The dose response following different running distances is also unknown, as is the recovery timeline for these different load profiles. A better understanding of how the proteoglycan concentration in healthy knee cartilage is altered by different running distances and how these alterations are alleviated over time is critical for determining safe exercise protocols and may serve as a foundation for future investigations targeting individuals suffering from cartilage-related conditions and altered mechanics.

Thus, the purpose of this study was to quantify changes to the biochemical state of femoral, tibial, and patellar cartilage before, immediately after, and 24 hours after running both 3 and 10 miles (4.8 and 16.1 km) in a group of young, asymptomatic, male runners. Exercise-induced biochemical changes in knee cartilage have been previously quantified using both T1rho and T2 relaxation mapping^3,6,20–22^. In this study, quantitative T1rho relaxation mapping was used to investigate these changes. T1rho relaxation times have been shown to be inversely correlated with the relative proteoglycan concentration in cartilage^22–25^. Specifically, water exudation results in increases in the relative proteoglycan concentration of articular cartilage, which subsequently leads to decreased T1rho values^22,26^. In particular, proteoglycan concentration, and thus T1rho relaxation times, has been shown to change following running^3,6^. Notably, Luke *et al*. measured elevated knee cartilage T1rho relaxation times both 48 hours and 3 months after running a marathon^3^, while Subburaj *et al*. revealed that knee cartilage T1rho relaxation times significantly decreased immediately after 30 minutes of treadmill running^6^. The present study builds upon the existing literature by investigating the dose effect and the recovery response to moderate running distances.

We hypothesized that both 3 and 10 mile runs would induce decreases in knee cartilage T1rho relaxation times, with larger changes occurring following the 10 mile run as compared to the 3 mile run. Specifically, we postulated that we would observe decreased T1rho relaxation times due to an increased relative proteoglycan concentration in knee cartilage post-exercise, which suggests water is exuded from the tissue during loading. Furthermore, due to fluid recovery into the cartilage, we hypothesized that the T1rho values measured 24 hours post-exercise would be significantly larger than those measured immediately post-exercise, approaching the baseline pre-exercise values.

## Methods

### Demographics

Eight healthy male runners (mean age: 31 years, range: 27–40 years; mean body mass index (BMI): 23 kg/m^2^, range: 18–25 kg/m^2^) were recruited to participate in this Duke University Institutional Review Board-approved study. All research methods were performed in accordance with these approved guidelines, and informed consent was obtained prior to enrollment. Individuals had no history of pain, injury, or surgery to the lower extremity, and all participants reported running a minimum of 5 miles (8 km) per week, on average, prior to the study.

### MRI and exercise protocol

**Figure 1:**
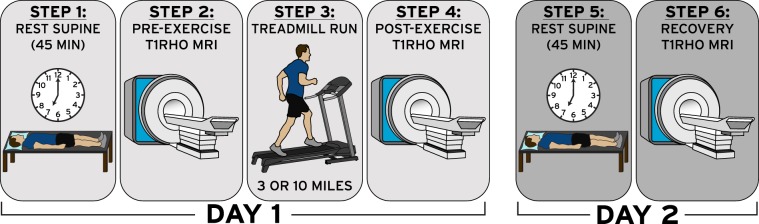
Workflow diagram of data collection sessions. This protocol was carried out in its entirety two times (on separate days) to test the effect of running 3 and 10 miles (4.8 and 16.1 km). Participants ran 10 miles on a treadmill during their first visit at a self-selected pace. The average pace from the 10 mile run was used approximately 2–3 weeks later when the individuals returned to run 3 miles.

To decrease the impact of diurnal variations on knee cartilage, individuals arrived at 7 am for all testing sessions **(**Fig. 1**)**^26–28^. Additionally, all participants were instructed to refrain from strenuous activity in the 24 hours prior to each visit, and they rested supine for 45 minutes to enable cartilage equilibration before baseline MRI^29,30^. Each subject’s dominant leg was determined based on the leg preferred to kick a ball^31^. Next, the knee on each participant’s dominant leg (7 right; 1 left) was imaged using a 3.0 T magnetic resonance (MR) scanner (Trio Tim; Siemens Medical Solutions USA; Malvern, PA) and an eight-channel knee coil (Invivo; Gainesville, FL). All scans were reviewed by a fellowship-trained musculoskeletal radiologist with over 30 years of experience in the field (C.E.S.) to confirm a lack of chondral abnormalities. Quantitative T1rho-weighted MR images were obtained using a spin-lock preparatory pulse followed by a 3D gradient recalled echo (GRE) pulse sequence (orientation: sagittal; field of view (FOV): 14 × 14 cm; matrix size: 256 × 128 pixels, interpolated to 256 × 256 pixels; resolution: 0.5 × 1.1 × 3.0 mm, interpolated to 0.5 × 0.5 × 3.0 mm; bandwidth: 130 Hz/pixel; flip angle: 15°; repetition time (TR): 3500 ms; echo time (TE): 5.9 ms; spin-lock frequency: 500 Hz; spin-lock times (TSLs): 5, 10, 40, 80 ms; acquisition time: 12 minutes, 30 seconds)^22,23,25,26,32^.

Following pre-exercise imaging, the individuals were transported via wheelchair approximately 10 meters to a treadmill in the room adjacent to the MR scanner. Each participant was instructed to set the treadmill at a pace at which they could run 10 miles. At the conclusion of the run, the participants were transferred via wheelchair back to the MR scanner for post-exercise imaging. An additional pulse sequence was run prior to acquiring the T1rho images, which delayed the acquisition by approximately 10 minutes.

Following the post-exercise scan, the individuals were instructed to refrain from strenuous activity for the rest of the day. Each participant returned the next morning at 7 am for a 45 minute rest period, before undergoing T1rho MR imaging again on their dominant knee. This process was repeated approximately 2–3 weeks later for a 3 mile run; however, instead of running at a self-selected pace, the treadmill was set to the average mile pace from each participant’s 10 mile run.

### 3D T1rho analysis

**Figure 2:**
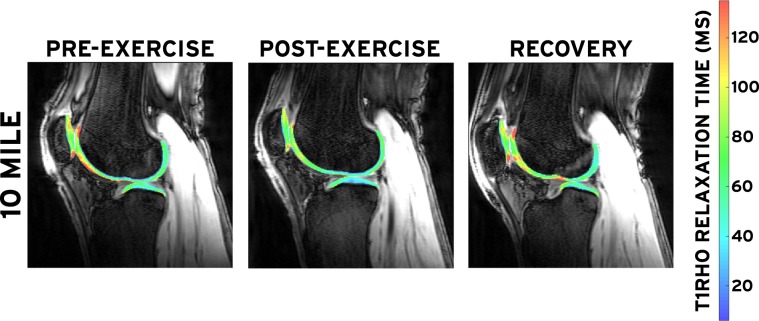
T1rho maps for a single participant before, immediately after, and 24 hours after running 10 miles (16.1 km). Red and blue are indicative of regions with high and low T1rho relaxation times, respectively.

The T1rho-weighted MR images were imported into custom image processing software (MATLAB; The MathWorks, Inc.; Natick, MA). First, the images were combined into a three-dimensional (3D) image stack for each spin-lock time. Then, the TSL = 10, 40, and 80 ms image stacks were individually rigidly registered about six degrees of freedom to the TSL = 5 ms stack, which was kept stationary. Next, voxels containing femoral, tibial, and patellar cartilage were manually selected in each TSL = 5 ms image by a single investigator (W.A.R.S.). These voxels were tracked across all spin-lock times, and the signal intensities were used in an exponential decay model (*S*(*TSL*) = *S*_0_exp(−*TSL*/*T*_1__*ρ*_)) to compute the T1rho relaxation time within each voxel, where *S*(*TSL*) is the signal intensity at each spin-lock time (TSL), *S*_0_ is the maximum signal intensity, and *T*_1__*ρ*_ is the T1rho relaxation time **(**Fig. 2**)**^32^. The computed T1rho values for all voxels within a single cartilage surface were averaged across all slices to determine the overall T1rho relaxation time corresponding to each MR scan (pre-exercise, post-exercise, and recovery). This technique has been shown to be repeatable in capturing mean baseline tibiofemoral cartilage T1rho values on separate days following a 45 minute rest period (coefficient of variation = 1.4%)^32^.

### Statistical analysis

An a-priori sample size estimation was based on previous investigations which quantified significant percent decreases in T1rho relaxation times following 20 minutes of treadmill walking (n = 6)^22^ and diurnal loading (n = 7)^26^. Assumptions of normality and the potential presence of outliers were examined by visual kernel density plots of residuals and by examining the inner and outer fences of the interquartile ranges of the residuals, respectively. A repeated measures analysis of variance (ANOVA) was used to determine how the independent variables bone type (femur, tibia, and patella), running distance (3 and 10 miles) and time point (pre-exercise, post-exercise, and recovery) impacted mean T1rho relaxation times (dependent variable). A separate repeated measures ANOVA was used to determine how bone type (femur, tibia, and patella) and running distance (3 and 10 miles) impacted post-exercise percent decreases in T1rho relaxation times. Additional analyses were performed to investigate compartment (medial vs. lateral vs. overall) as an independent variable (see Supplementary Information). Significant ANOVA results were followed up with Fisher’s Least Significant Difference (LSD), which is an appropriate post-hoc test after an ANOVA with three comparisons or less^33^. Results are reported as the mean ± standard error of the mean (SEM). Alpha was set as p < 0.05 for all analyses. All statistical analyses were performed using Stata 16.0 (StataCorp LLC; College Station, TX) and Statistica (TIBCO Software, Inc.; Palo Alto, CA), and they were overseen by an experienced epidemiologist with expertise in biostatistics (A.P.G.).

## Results

**Figure 3:**
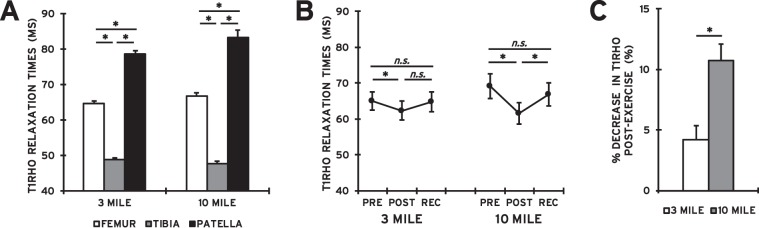
(**A**) Mean (±SEM) femoral, tibial, and patellar cartilage T1rho relaxation times in response to 3 and 10 mile runs (4.8 and 16.1 km). For each running distance, patellar cartilage T1rho values were significantly larger than femoral cartilage T1rho relaxation times, and both patellar and femoral cartilage T1rho values were significantly greater than tibial T1rho values (*p < 0.001). **(B)** Mean (±SEM) T1rho relaxation times before (PRE), immediately after (POST), and 24 hours after (REC) running 3 and 10 miles (averaged across all bones). T1rho relaxation times significantly decreased immediately post-exercise for both 3 (*p = 0.04) and 10 (*p < 0.001) mile runs. No differences were observed between the pre-exercise and recovery values for both 3 (p = 0.8) and 10 (p = 0.08) mile runs. **(C)** Percent decreases in knee cartilage T1rho relaxation times (mean ± SEM) immediately post-exercise (averaged across all bones). Significantly greater percent decreases in T1rho relaxation times were observed after the 10 mile run as compared to the 3 mile run (*p = 0.02).

On average, the subjects ran 3 and 10 miles in 0:29:18 ± 0:00:56 and 1:37:28 ± 0:03:05 (h:mm:ss), respectively. This corresponds to a mean mile pace of 0:09:45 ± 0:00:19. An average of 21 ± 2 days elapsed between the 10 mile and 3 mile runs. A repeated measures ANOVA showed significant interactions between bone type and distance (p = 0.009), as well as time point and running distance (p = 0.04) on T1rho relaxation times **(**Table 1**)**. The bone type × distance interaction showed that the mean femoral (3 mile: 65 ± 1 ms; 10 mile: 67 ± 1 ms), tibial (3 mile: 49 ± 1 ms; 10 mile: 48 ± 1 ms), and patellar (3 mile: 79 ± 1 ms; 10 mile: 83 ± 2 ms) cartilage T1rho relaxation times were significantly different from each other for both running distances (p < 0.001; Fig. 3A). Furthermore, the time point × distance interaction demonstrated that mean T1rho values significantly decreased immediately after running 3 (65 ± 3 ms to 62 ± 3 ms; p = 0.04) and 10 (69 ± 4 ms to 62 ± 3 ms; p < 0.001) miles **(**Fig. 3B**)**. No significant differences were observed between the 24 hour recovery T1rho relaxation times and their corresponding pre-exercise values after both 3 (65 ± 3 ms vs. 65 ± 3 ms; p = 0.8) and 10 (69 ± 4 ms vs. 67 ± 3 ms; p = 0.08) mile runs **(**Fig. 3B**)**. While the 10 mile recovery T1rho values were significantly greater than the corresponding post-exercise values (p < 0.001), this was not the case for the 3 mile run (p = 0.07; Fig. 3B). Additionally, the second ANOVA illustrated a significant main effect of distance (p = 0.02, Table 2), with larger percent decreases in T1rho relaxation times following 10 mile runs as compared to 3 mile runs (11 ± 1% vs. 4 ± 1%; Fig. 3C).

**Table 1:** Three-Way Repeated Measures ANOVA (T1rho Relaxation Times).

	Variables	p-Value
Main Effects	Bone (Femur/Tibia/Patella)	<0.001*
	Time Point (Pre/Post/Rec)	<0.001*
	Distance (3 mile/10 mile)	0.048*
Interactions	Bone × Time Point	0.645
	Bone × Distance	0.009*
	Time Point × Distance	0.043*
	Bone × Time Point × Distance	0.668

*p < 0.05.

**Table 2:** Two-Way Repeated Measures ANOVA (% Decrease in T1rho Relaxation Times).

	Variables	p-Value
Main Effects	Bone (Femur/Tibia/Patella)	0.409
	Distance (3 mile/10 mile)	0.022*
Interaction	Bone × Distance	0.487

*p < 0.05.

## Discussion

In this study, we demonstrated that femoral, tibial, and patellar cartilage experience statistically significant percent decreases in T1rho relaxation times immediately following both 3 and 10 mile runs, with significantly larger changes occurring following 10 mile runs as compared to 3 mile runs. The results also show that knee cartilage T1rho relaxation times were able to recover to within 2% of their baseline values within 24 hours of running up to 10 miles. The results of this investigation provide baseline data for young, asymptomatic, male runners that may be used in the future to help guide the development of safe exercise and recovery protocols. Additionally, the quantitative MRI and exercise framework employed in this study may also be used in the future with symptomatic populations to evaluate the incidence of running-related patellofemoral pain^34^ and other cartilage-related conditions.

In the present study, we reported significant percent decreases of 4% and 11% in knee cartilage T1rho relaxation times following 3 and 10 mile runs, respectively. Our results are in agreement with previous investigations that have quantified how running and other activities impact both T1rho and T2 relaxation times^6,20–22,26,35^. Specifically, Subburaj *et al*. demonstrated a 9% reduction in knee cartilage T1rho immediately after 30 minutes of running^6^. Similarly, Hatcher *et al*. reported a 5% decrease in tibiofemoral cartilage T1rho relaxation times after 20 minutes of treadmill walking^22^, while Taylor *et al*. observed a 7% decrease in tibial cartilage T1rho values in response to activities of daily living^26^. Consistent with these findings, Gatti *et al*.^20^, Subburaj *et al*.^6^, and Behzadi *et al*.^21^ reported decreases in knee cartilage T2 relaxation times after 15, 30, and 45 minute runs, respectively. Specifically, our results not only indicate that knee cartilage T1rho relaxation times significantly decrease after 3 and 10 mile runs, but that the tissue is able to return to its baseline T1rho value within 24 hours post-exercise. While safe levels of cartilage loading are currently unknown, the similarities between our findings and those reported previously in response to walking^22^ and activities of daily living^26^ may suggest that moderate running distances (up to 10 miles) are within a safe, physiological range. Further studies are needed to substantiate this claim.

In contrast to the findings of this investigation, some studies have reported increased T1rho relaxation times following loading, which are indicative of a relative increase in water concentration in the tissue^22–25^. For instance, Luke *et al*. observed that knee cartilage T1rho values were elevated by 5% 48 hours after running a marathon^3^. As suggested by the present investigation, the prescribed loading magnitude and duration, as well as the time elapsed between loading and the MRI scans, likely play important roles in modulating T1rho relaxation times. Additionally, long-term cartilage damage is possible as a result of the marathon run^3^, which could degrade the proteoglycan concentration of the tissue and subsequently increase the measured T1rho relaxation times^22^. While it is difficult to directly compare studies due to methodological differences, these factors may help to explain the differences between our results and those obtained previously.

Importantly, the values reported in the current investigation (3 mile: 4%; 10 mile: 11%) may be underestimates of the maximal post-exercise decreases in T1rho relaxation times. Our study design required that we collect another MRI sequence prior to collecting the T1rho-weighted images. Though the precise recovery time course remains unclear, patellar cartilage has been previously shown to recover 50% of its fluid loss within 45 minutes of performing 100 deep knee bends^29^. Thus, the knee cartilage was likely recovering during the 10 minutes before and throughout the duration of the T1rho scan (12 minutes, 30 seconds), leading to an underestimate of the overall percent decrease in T1rho relaxation times.

Despite this, we observed a significant effect of distance on percent changes in T1rho relaxation times, with larger decreases in T1rho values following 10 mile runs as compared to after 3 mile runs. Cartilage deformations have been previously modeled by creep behavior, whereby steady-state is reached over time^36,37^. These findings might suggest that runs exceeding 10 miles in length could lead to even larger percent decreases in T1rho relaxation times, and the percent changes may eventually plateau as the running distance is increased. Specifically, Paranjape and Cutcliffe *et al*. reported increased tibial cartilage strains in response to increasing walk durations, and they noted a leveling off effect as the walking activity approached 60 minutes^37^. Future projects may seek to quantify the precise dose-response of the tissue to runs of different lengths to further investigate this phenomenon. Additionally, we did not detect statistically significant differences between pre-exercise and recovery knee cartilage relaxation times. These values significantly decreased immediately post-exercise prior to returning to within 2% of their pre-exercise T1rho relaxation times by the end of the 24 hour recovery period, but it is unclear exactly how long it took the cartilage to recover. Future studies may more closely probe the recovery timeline of the cartilage post-exercise, as this information may be critical for identifying optimal exercise routines.

The purpose of this study was to better understand how the biochemical composition of healthy knee cartilage is affected by different running distances, both immediately post-exercise and following a 24 hour recovery period. This study sought to include young, asymptomatic, male participants with no history of pain, injury, or surgery to the lower extremity. In the present investigation, only male runners were included, as previous studies have shown differences in tibiofemoral cartilage between males and females^38^. Future studies may investigate the effects of other variables, including age, sex, activity level^20^, and the presence of patellofemoral pain on these biochemical changes. Additionally, while joint loading during short duration runs has been previously shown to have a protective effect on cartilage^4,39^, longer runs over an extended period of time may be detrimental to cartilage health^3,40^. Therefore, while ideal cartilage loading is currently unclear, the current investigation demonstrated that knee cartilage was able to return to within 2% of its baseline T1rho relaxation time within 24 hours of running up to 10 miles, suggesting that this exercise may fall within the realm of healthy cartilage loading. Furthermore, in the context of overuse and injury, running can potentially alter the likelihood of developing osteoarthritis (OA) later in life; however, many other factors, including age, sex, BMI, and exercise type and intensity may also play an important role in OA development, making it difficult to determine a causal relationship^2,41–43^.

In conclusion, this study demonstrated that knee cartilage T1rho relaxation times significantly decrease after running both 3 and 10 miles, with larger decreases occurring in response to 10 mile runs as compared to 3 mile runs. After a 24 hour recovery period, the measured T1rho relaxation times recovered to within 2% of their pre-exercise values. This information serves as a baseline for asymptomatic male runners that may be used to help guide safe exercise and recovery protocols.

## Supplementary information

Supplementary Material.

## Data Availability

Data relevant to this work will be made available upon reasonable request.
