# Supplementary material for: Quantifying the biochemical state of knee cartilage in response to running using T1rho magnetic resonance imaging
Source: Sci Rep. 2020 Feb 5;10:1870. doi: 10.1038/s41598-020-58573-8 (PMC7002650; doi:10.1038/s41598-020-58573-8)
Supplement: Supplementary file 1 — Supplementary Material. [file 41598_2020_58573_MOESM1_ESM.pdf]

Quantifying the biochemical state of knee cartilage in response to running using T1rho magnetic resonance imaging

Lauren N. Heckelman[1,2], Wyatt A.R. Smith[1], Alexie D. Riofrio[3], Emily N. Vinson[3], Amber T. Collins[1], Olivia R. Gwynn[1,2], Gangadhar M. Utturkar[1], Adam P. Goode[1,4,5], Charles E. Spritzer[3], Louis E. DeFrate[1,2,6,*]

[1]Department of Orthopaedic Surgery, Duke University School of Medicine, USA

[2]Department of Biomedical Engineering, Pratt School of Engineering, Duke University, USA

[3]Department of Radiology, Duke University School of Medicine, USA

[4]Department of Population Health Sciences, Duke University School of Medicine, USA

[5]Duke Clinical Research Institute, USA

[6]Department of Mechanical Engineering & Materials Science, Pratt School of Engineering, Duke University, USA

***Corresponding author:**

Louis E. DeFrate, ScD

Duke University Box 3093

Durham, NC 27710 USA

Phone: (919) 681-9959

Email: lou.defrate@duke.edu

Supplementary Information

<u>Medial versus Lateral Compartmental Analysis</u>

Each cartilage region (femoral, tibial, and patellar cartilage) was also subdivided into medial and lateral compartments, and the mean T1rho relaxation times were computed for each sub-region. A repeated measures ANOVA was performed to investigate the impact of the independent variables of bone type (femur, tibia, and patella), time point (pre-exercise, post-exercise, recovery), distance (3 and 10 miles), and compartment (medial, lateral, and overall) on mean T1rho relaxation times. A separate, analogous repeated measures ANOVA was also used to investigate how bone type, distance, and compartment influence immediate post-exercise percent decreases in T1rho relaxation times. Significant effects of compartment (medial vs. lateral) were not observed in either analysis **(Supplementary Tables S1 & S2)**. In fact, both the medial and lateral compartment T1rho relaxation times similarly decreased immediately post-exercise and recovered within 24 hours **(Supplementary Figure S1)**. Therefore, this medial versus lateral division did not impact the interpretation of our primary analysis.

Supplementary Table S1. Four-Way Repeated Measures ANOVA (T1rho Relaxation Times)

	Variables	p-Value
**Main Effects**	Bone (Femur/Tibia/Patella)	**<0.001***
	Time Point (Pre/Post/Rec)	**<0.001***
	Distance (3 mile/10 mile)	0.061
	Compartment (Medial/Lateral/Overall)	0.075
**Interactions**	Bone × Time Point	0.618
	Bone × Distance	**0.008***
	Time Point × Distance	**0.037***
	Bone × Compartment	0.081
	Time Point × Compartment	0.668
	Distance × Compartment	0.329
	Bone × Time Point × Distance	0.675
	Bone × Time Point × Compartment	0.827
	Bone × Distance × Compartment	0.863
	Time Point × Distance × Compartment	0.377
	Bone × Time Point × Distance × Compartment	0.866

*p<0.05

Supplementary Table S2. Three-Way Repeated Measures ANOVA (% Decrease in T1rho Relaxation Times)

	Variables	p-Value
**Main Effects**	Bone (Femur/Tibia/Patella)	0.463
	Distance (3 mile/10 mile)	**0.023***
	Compartment (Medial/Lateral/Overall)	0.689
**Interactions**	Bone × Distance	0.523
	Bone × Compartment	0.991
	Distance × Compartment	0.218
	Bone × Distance × Compartment	0.870

*p<0.05

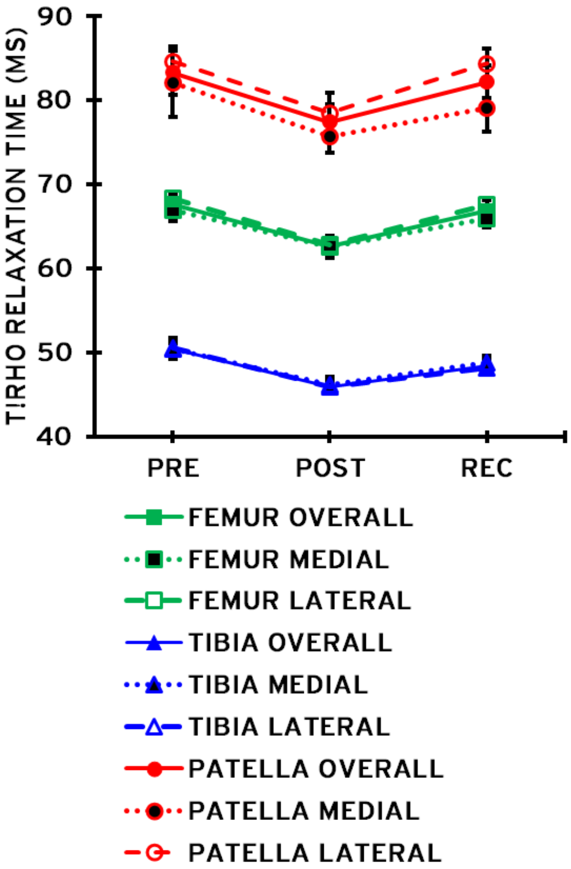

Supplementary Figure S1. Mean (±SEM) femoral, tibial, and patellar cartilage T1rho relaxation times at baseline (PRE), immediately post-exercise (POST), and following a 24 hour recovery period (REC), subdivided into medial and lateral compartments. No significant effects of compartment were observed.
